# Mechanical and Tribological Behavior of Functionally Graded Unidirectional Glass Fiber-Reinforced Epoxy Composites

**DOI:** 10.3390/polym14102057

**Published:** 2022-05-18

**Authors:** Waleed Alhazmi, Yosef Jazaa, Sultan Althahban, Saeed Mousa, Ahmed Abu-Sinna, Amr Abd-Elhady, Hossam El-Din Sallam, Mahmoud Atta

**Affiliations:** 1 Faculty of Engineering, Jazan University, Jazan 706, Saudi Arabia; smalthahban@jazanu.edu.sa (S.A.); samousa@jazanu.edu.sa (S.M.); 2 Faculty of Engineering, King Khalid University, Abha 61421, Saudi Arabia; yjazaa@kku.edu.sa; 3 Force and Material Metrology Department, National Institute of Standards, Giza 12211, Egypt; ahmed.abusinna@nis.sci.eg; 4 Mechanical Design Department, Faculty of Engineering, Helwan University, Cairo 11718, Egypt; amr_abdelfattah@m-eng.helwan.edu.eg; 5 Faculty of Engineering, Zagazig University, Zagazig 44511, Egypt; hem_sallam@zu.edu.eg (H.E.-D.S.); matta767@gmail.com (M.A.)

**Keywords:** functionally graded material, mechanical properties, small punch test, tribological properties, 3D-FEA

## Abstract

This paper aims to assess experimentally the mechanical and tribological behavior of conventional and functionally graded (FG) polymeric matrix composites reinforced with continuous glass fibers. The small punch test (SPT) and a pin-on-disc device were used in the present work to examine the mechanical and wear behavior, respectively. The hand lay-up technique was used in the present investigation to manufacture the conventional and FG composites. Various wooden looms with different nailed spacing were employed to manufacture the FG composites. According to test type, the FG composite is composed of four and ten layers, with a different glass fiber volume of fraction (*V_f_*%) for each layer. In addition, the finite element simulation based on Hashin’s failure criterion and cohesive zone modeling was used to show the progressive failure and give more explanation regarding the flexural behavior of such composites. The present results indicate that the wear rate of an FG composite could be affected by many factors, including the disk speed, applied load, the composite layers number, and average glass fiber volume fraction. On the other hand, the arrangement of layers in the composite materials by variation of *V_f_*% for each layer can improve the wear rate and value of the ultimate load before the fracture of the composite material when subjected to SPT. The experimental and numerical results for all SPT specimens showed that the fracture of the SPT specimens began beneath the punch tip and grew along the fiber direction. The ultimate flexural capacity of FG composites increased by 30% compared with the conventional composites.

## 1. Introduction

Recently, polymer composite materials have been used in manufacturing and many industrial applications. The low cost, the ease of maintenance, and the improved strength are the main reasons for their selection. Despite these advantages, some serious disadvantages are presented, such as delamination due to heavy work or extreme thermal loads [1]. Functionally graded materials (FGMs) were introduced as an advanced method to overcome this drawback. FGMs can be described as a distinct approach to obtaining the best features at the lowest cost and specific desired [2]. The material properties of FGMs vary through the graded direction of a structure [3]. FGM is a novel production method used in the medical, aerospace, and industrial fields for applications such as wear-resistant coatings, cutting tools, and biomedical devices [4,5,6]. FGMs can enhance product properties better than conventional composite materials. Single-fiber reinforced FGMs exhibit more outstanding properties than conventional hybrid composites [7]. Properties like hardness, wear resistance, toughness, specific strength, modulus of elasticity, and weight can be improved by using FGM [8,9,10,11,12,13,14,15]. FGMs are not limited to polymers, as it also has applications in ceramics and metallic materials, especially tribology applications [16].

Investigations have concluded that speed, sliding and sliding time, applied load, thermal conditions, radial distance, and reinforcement type are the most influential parameters in the tribological behavior of wear abrasives [16]. Loads of 28 N, 45 N, 63 N, and 80 N were applied to abrasives tests conducted by Radhika et al. The effect of increasing the loads was investigated [17]. The specimen surface was worn out with fewer surface cuts and ploughings under the lowest load condition. The silica sand produced mild abrasions with a slightly higher load and more surface cuttings. The entrance zone contained crushed sand particles that produced a tribo-system, accompanied by vibration by increasing the load, creating deep grooves and severe cutting wear. A combination of erosive wear, grooving, and abrasion with severe wear materials appeared when using the highest selected loads. The previous study was not limited to investigating only the effect of load, as speed was also investigated. Speeds of 50 r/min, 100 r/min, 150 r/min, and 200 r/min were chosen in the study to investigate the wear rate behavior [17]. The results showed an inverse relationship between the speed and abrasive wear rates. The time of contact between the silica sand and the specimen decreased when increasing the speed rate, leading to a decreasing wear rate [17,18]. Gautam et al. used an FGM containing vinyl ester resin as a matrix material and its respective hardener, reinforced with E-milled glass fiber and graphite, in their research [19]. Results showed an increase in wear rate per unit load related to the increase in the normal load and sliding distance.

A comparison between the homogeneously distributed fiber composites and FGMs fiber composite was conducted by Gangil et al. [20]. The results showed the superiority of the FGM fiber composite over the homogenously distributed fiber composites. Additionally, increasing the fiber volume fraction enhanced the mechanical properties compared to the homogeneous composite [21]. When using FGM reinforced with chopped carbon fibers, the results showed that the significant effect factors used in the wear test were the medium of the abrasives and the fiber fraction. In contrast, the lowest effect was seen in the normal load [22]. Another comparison between the homogenous fiber distribution and the FGM was conducted. A fixed Kevlar® fiber (5 wt.%) with varying bagasse fiber (0, 5, 10, and 15 wt.%) was used to reinforce vinyl ester composites for homogeneous and FGMs. Four variables were used to evaluate the sliding test behavior (fiber content, normal load, sliding distance, and sliding velocity). The results concluded that the largest factor affecting this test was the normal load for the two composites. This conclusion is opposite to the previous study’s conclusion, the reason for which might be explained by different reinforcements.

A study using a composite of unidirectional oriented E-glass fiber-reinforced epoxy was conducted using the pin-on-ring technique to investigate the friction and wear properties. The results reflect that increasing sliding under clean or wet conditions improves the friction coefficient and wear rate [23,24]. The ball-on-disk technique was used to study the tribological properties of FGM reinforced by carbon nanotubes (CNT), which demonstrated increasing coefficients of friction and support abrasion resistance with adding CNT [25].

Small punch testing (SPT) is considered one of the best techniques to obtain a material’s mechanical properties. The design of the SPT is based on the idea of subjecting a thin test sheet to a combination of both bending and stretching. The test setup consists of a die with upper and lower parts to fix the position of the specimen and a punch with a spherical tip to penetrate the specimen under constant speed. Recently, SPT has been widely used to estimate the mechanical properties of metallic and nonmetallic materials, especially when there is no excess of materials [26,27,28,29,30].

M Atta et al. [31] used the 3D finite element simulation to predict the flexural strength of conventional and FGM based on Hashin’s failure criterion. Hirshikesh et al. provided a view into the crack growth resistance of FGMs [32]. They benchmark their predictions with results from alternative numerical methodologies. The results prove the capability of their model in the areas of predicting crack initiation from arbitrary nucleation sites, capturing crack deflection without remeshing, and modeling unstable crack propagation in an implicit framework. Abd-Elhady et al. [33] used a Hashin damage model to predict the damage failure of a pinned joint in quasi-isotropic laminates. The effect of a bonded polymeric composite patch on reducing the crack tip driving forces of a cracked metallic element under cyclic loading was simulated numerically by Abd-Elhady [34,35].

FGMs present a good choice when focusing on lighter weight and a higher strength in advanced composite materials when compared to conventional composites. The basic idea of the present FGM study is to propose a potential alternative to the standard “glass skin/PVC foam core” sandwiches, which are widely used in the transport industry [31]. The main objective of the present work is to improve the mechanical and tribological behaviors of polymeric composites reinforced with continuous glass fibers by using the FGM technique. The mechanical and tribological behaviors are determined using a small punch test and pin-on-disc device. For further understanding, the finite element (FE) simulation based on Hashin’s failure criterion and cohesive zone modeling (CZM) is adopted. The present work is considered a continuation of the author’s previous work [31] to obtain a complete analysis for functionally graded unidirectional glass fiber-reinforced epoxy composites material. The novelty of the present work lies in adopting SPT to compare the flexural strength of FG with conventional composites. The effect of FG patterns on the wear resistance of polymeric fibrous composites represents the originality of this research.

## 2. Experimental Works

This section deals with the hand lay-up manufacturing technique and the instruments and equipment used in measuring the mechanical and tribological behavior of the FGM. The hand lay-up manufacturing technique was used to prepare the conventional and functionally graded composite materials using unidirectional glass fiber/epoxy. Furthermore, the small punch test and a pin-on-disc device were used to examine the mechanical and wear behavior of the FGM, respectively. However, two primary specimen shapes have been manufactured according to the wear and small punch tests (SPT).

### 2.1. Material Preparation and Mechanical Properties

**Figure 1:**
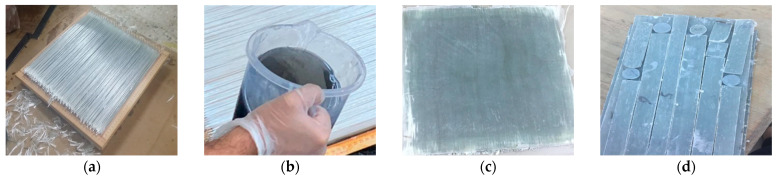
Stages of the manufacturing of the epoxy/fiberglass FGM; (**a**) loom with unidirectional fiberglass bundles, (**b**) pouring epoxy over the fiberglass layers, (**c**) the FGM product, and (**d**) manufactured wear and SPT specimens.

The constituent materials of conventional and functionally graded composite material are epoxy, hardener, and fiberglass. The mechanical properties of the materials used are listed in Table 1. Figure 1 shows the steps of manufacturing conventional and functionally graded composite materials using wooden looms with nailed spacing. Nine sets of looms were selected and arranged vertically to make either four or ten layers of parallel fiberglass strings, as shown in Figure 1. The difference between layers is the loom nailing space which directly influences the glass fiber volume of fraction for each layer, *V_f_*, to compose the FGM, as shown in Table 2. The specimens were named according to the glass fiber volume fraction for each layer, *V_f_*, used in the manufacturing. The glass fiber volume of fractions for each layer, *V_f_*; the values are graded experimentally using ignition loss testing conducted based on ASTM D 3171-15 [36]. The volume fractions, generated due to different nail spacings for different groups, which are to be used either in wear or SPT tests, are listed in Table 2. All SPT specimens were formed using four layers of FGM to fulfill the dimensions of the testing space of the SPT setup.

### 2.2. Sample and Tests Characterization

#### 2.2.1. Wear Tests

**Figure 2:**
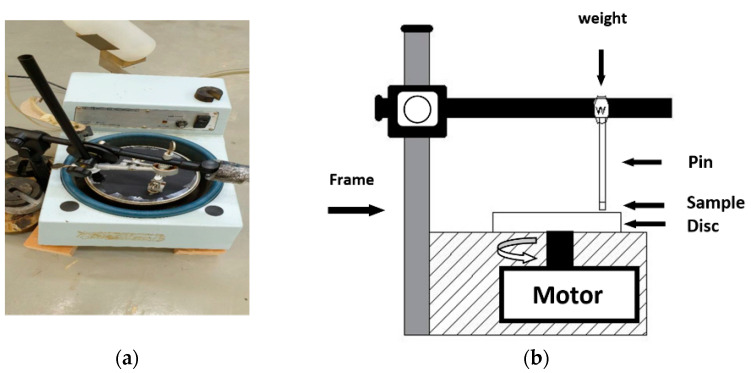
(**a**) Custom-built tribometer used in this study; (**b**) diagrammatic sketch of the wear test setup.

Tribological experiments are conducted according to ASTM F732 standards [37] to determine conventional and functionally graded composite materials’ wear behavior using a pin-on-disk machine, as shown in Figure 2. In the wear tests, each sample was fixed on a pin and then sited on a new bare-wood sandpaper disk with a grit size of 220 for each test, at a fixed distance from the disk center. In the wear test, the disk used as the abrasive counter-face was rotated at three speeds, 0.39, 0.79, and 1.5 m/s, for one minute in order to check the effect of the variable speed on the composite material wear. Likewise, two forces were applied to the specimens, namely 30 N and 50 N. The square specimen was used in the wear test with a length of 18 mm, and an average thickness of 8 mm. The relative wear rate percentage was measured in terms of the wear weight loss, based on the method reported by Czichos et al. [38]

Five assemblies of looms were designated for the wear test, namely, *M*_1_, *M*_2_, *M*_3_, *M*_4_, and *M*_5_, as shown in Table 2, where the average glass fiber volume fractions, *V_fa_*. *M*_2_ and *M*_3_, are composed of ten layers with varying glass fiber volume fractions for each layer, in order to understand the tribological behavior of the FGM. Furthermore, two conventional composite materials formed by ten layers, M1 and M4, with a uniform glass fiber volume of fraction for each layer, were assigned to be compared with the FGM specimens. Lastly, the *M*_5_ specimen made of conventional composite materials was formed by four layers and had an average glass fiber volume of fraction of *M*_4_ and was used to study the effect of layers number on the tribological behavior of composite materials.

#### 2.2.2. Small Punch Tests (SPT)

**Figure 3:**
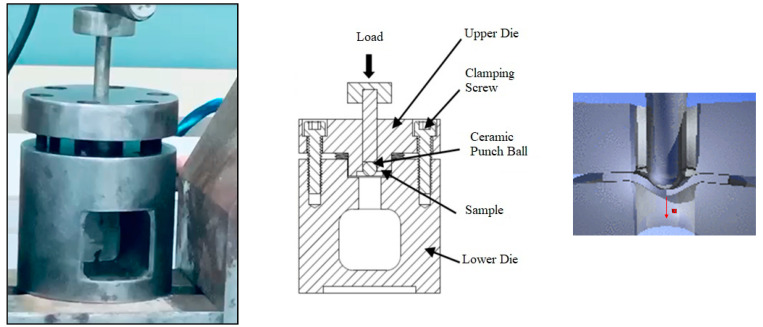
Small punch test setup.

The small punch (SP) technique for testing small specimens has received much interest since its introduction in the 1980s in fusion and fission programs for structural alloys, mainly in the U.S. and Japan. The SP test is a displacement-controlled testing process, i.e., the punch is pushed with a constant velocity of the crosshead through the specimen, and the force required to keep the punch moving is measured as a function of punch displacement (at the punch tip) or specimen deflection *u* (measured on the lower side of the specimen, opposite the contact point between the punch and the specimen) [39]. In Figure 3, a photo shows the SP set and a schematic drawing representing the punching test.

Four sets of looms were designated experimentally for the SPT process, namely, *M*_6_, *M*_7_, *M*_8_, and *M*_9_, as shown in Table 2, each with a different average glass fiber volume fraction, *V_fa_*. *M*_6_ and *M*_8_ were conventional composite layers assigned as references, while *M*_7_ and *M*_9_ were assigned for FGM samples. Therefore, the total number of layers used in the SPT specimen is four layers in each specimen, as shown in Table 2. The geometry of the SPT specimen is 20 mm in diameter, with an average thickness is 3.9 mm.

## 3. Finite Element Model for the SP Test

Modeling the SPT test was used to confirm the test results. This allows additional studies for more specimen configurations to study the advantages of the FGMs over the conventional composite materials. Four additional specimens, labeled *M*_10_, *M*_11_, *M*_12_, and *M*_13_ were used as a parametric study, as shown in Table 3. Whereas *M*_10_ and *M*_11_ have a constant average *V_fa_* = 30% with variation in *V_f_* of the layers in comparison to its mechanical properties with that of *M*_8_, while *M*_10_ and *M*_11_ have a constant average *V_fa_* = 20% with variation in *V_f_* of the layers when comparing its mechanical properties with that of *M*_6_. To model the failure of the four layers of epoxy composite reinforced with E-glass through the SPT, 3D FE analysis was utilized. The specimen was constructed with four layers, with the appropriate *V_f_* for each layer. The layers were bonded to each other using an adhesive layer. The failure through the current simulation is based on the Hashin damage criterion and CZM by using ABAQUS/Standard codes [40] to predict the failure due to fiber breakage, matrix cracking, and delamination in composite layers. The Hashin damage criterion was used to predict the mode of intra-laminar damage based on Hashin’s theory [41,42], which considers four different failure modes: fiber tension, fiber compression, matrix tension, and matrix compression. Otherwise, CZM is used to simulate the cohesion between the layers. A thin adhesive layer of epoxy was formed. CZM is based on the traction separation law. The traction separation law assumes initial linear elastic behavior followed by a linear damage evolution. The maximum nominal stress criterion, which is determined by the adhesive material properties such as modulus of elasticity (*E*) and shear modulus (*G*), has been adopted to determine the damage initiation.

The elastic constants and Hashin’s damage model strength data of the different layers, according to their *V_f_*%, are shown in Table 4 and Table 5, respectively, extracted analytically as was described in refs. [31,43]. CZM was used to simulate the adhesive layers. The CZM relies on the traction separation law with elastic ratios *E/E_nn_*, *G*_1_*/E_ss_*, and *G*_2_*/E_tt_* calculated with equations (1 to 3) [40,44] with nominal stress 61 MPa.

*E*/*E_nn_* = *K*_1_ = (*E* × *α*)/*t* (1)

*G*_1_/*E_ss_* = *K*_2_ = (*G*_12_
*×*
*α*)/*t* (2)

*G*_2_/*E_tt_* = *K*_3_ = (*G*_13_
*×*
*α*)/*t* (3)

where *E* is the epoxy’s modulus of elasticity, *G* is the Shear modulus of epoxy, *α* is a constant = 50, and *t* = cohesive layer thickness. For more details, refer to Sec-1: Mechanical properties of glass fiber-epoxy lamina; Sec-2: Hashin Model Constants; Sec-3: Hashin Damage Criteria; Computer Code: Computer Code.inp (see Supplementary Materials) [31,40,43,45].

**Figure 4:**
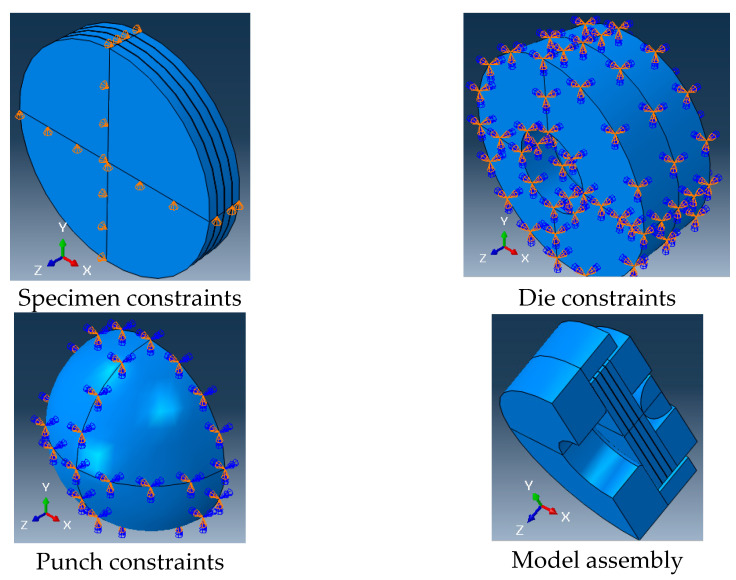
Constrains and assembly of the different elements of the FE model.

Contact between the composite and cohesive layers was assumed to be tie contact (perfect bonding). Meanwhile contact between the outer composite layers of the test specimen with a punch, the upper die, and the lower die was made using a master–slave algorithm (surface-to-surface contact type) in ABAQUS [40]. The specimen’s outer surfaces were considered to be slave surfaces. The contact between the metallic parts and the composite layers was simulated as penalty friction with a friction coefficient of 0.3 [46,47]. Both parts of the die were set to have no degrees of freedom. The punch had only one degree of freedom in the z-axis, as shown in Figure 4. The center planes of the specimen had five degrees of freedom (the YZ plane was constrained in the x-direction, while the XZ plane was constrained in the y-direction), as seen in Figure 4.

**Figure 5:**
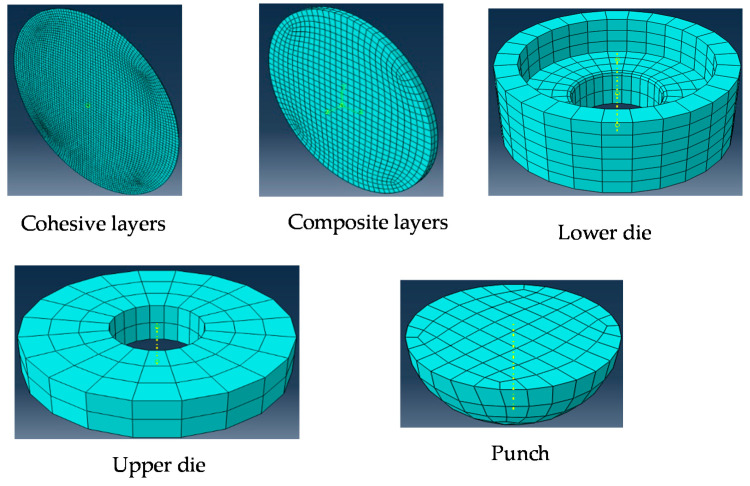
Mesh of the different elements of the FE model.

**Figure 6:**
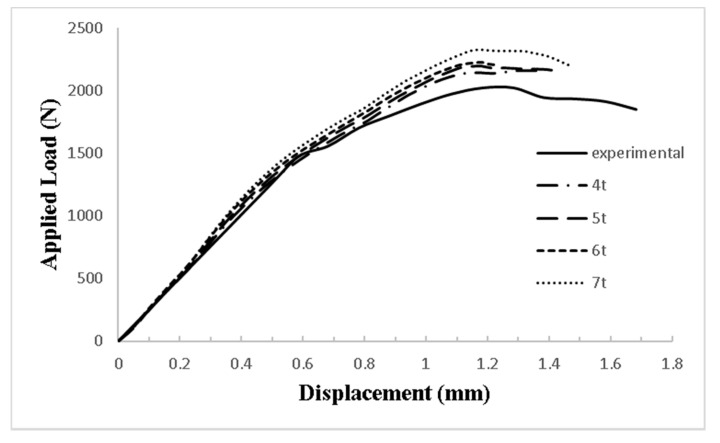
The effect of the size of the cohesive layer elements in the FE model results (where *t* is the thickness of the cohesive layer).

The current modeling analysis simulated the die (two parts) and the punch with linear brick elements (eight nodes, C3D8R elements). The composite specimen layers were represented by 8-node quadrilateral continuum shell elements (SC8R), which is compatible with Hashin’s damage model. An 8-node three-dimensional cohesive element (COH3D8) was used to model the cohesive layers. Figure 5 shows the mesh of the different elements of the finite element model. The mesh element size of the composite layers was taken as 0.7 mm [31]. Figure 6 shows the effect of the cohesive elements’ size in the model results. The figure shows that the most suitable element size for the cohesive layer was five times its thickness, *t*.

## 4. Results and Discussion

### 4.1. Wear Results

**Figure 7:**
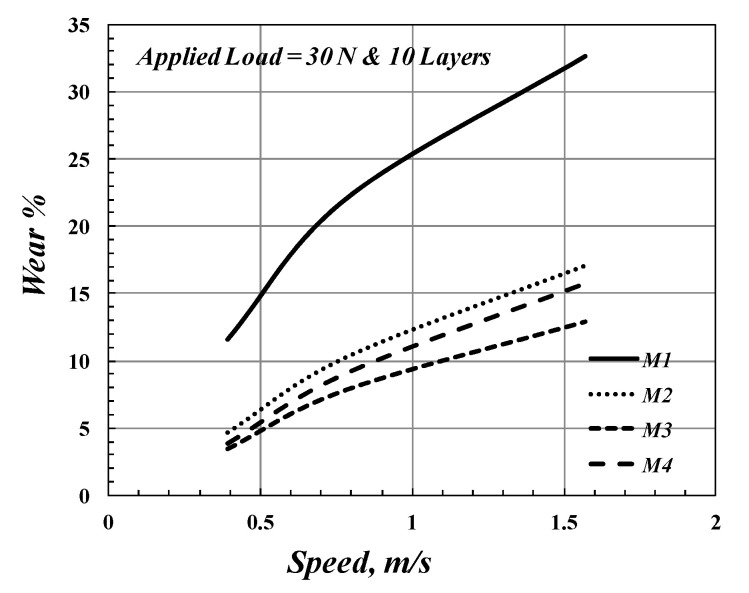
The effect of the speed on the wear rate.

The sliding wear test produced abrasive wear on the sample’s surface. Furthermore, it can be seen that the maximum wear rate is observed for *M*_1_, which has zero fiber, an average glass fiber volume of fraction, *V_fa_*. The variation of the wear rate of the FGM at the various speeds is shown in Figure 7. It is clearly seen that the wear rate increases when increasing the disk speed, as shown in Figure 7. This phenomenon is caused by the deteriorating adhesion between the fibers and matrix. On the other hand, the wear rate of composite materials decreases with increasing fiber fractions. This phenomenon is attributed to various factors, including the presence of glass fibers, which can strengthen the matrix and reduce the load on the surface.

**Figure 8:**
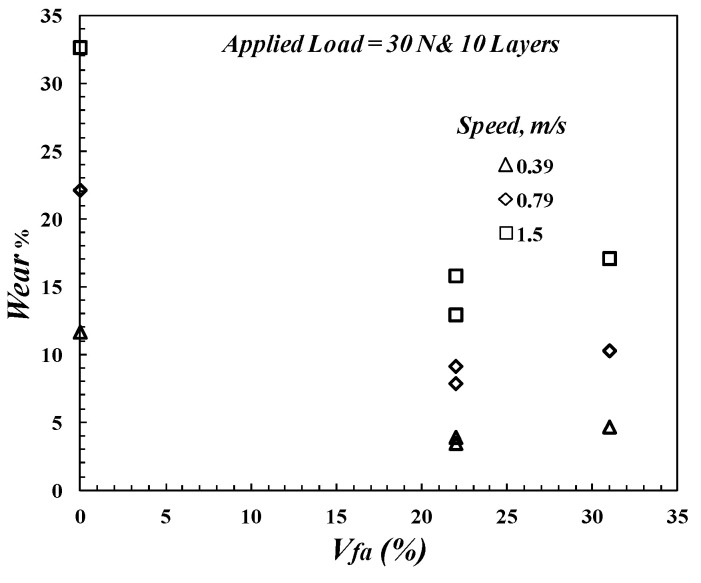
Wear rate as a function of average glass fiber volume of fraction, *V_fa_*.

On the other hand, the wear rate decreases when increasing the *V_fa_*. Simultaneously, the arrangement of the layers of FGM based on the *V_f_* of each layer affects the wear rate, as shown in Figure 7. In other words, specimen *M*_3_ has a lower wear rate than *M*_4_, although they have the same *V_fa_*. However, the *M*_2_ specimen (which has a *V_fa_* of 31%) has a higher wear rate than the *M*_3_ specimen (which has *V_fa_* of 22%). Figure 8 clearly describes the effect of average glass fiber volume of fraction (*V_fa_*) on the wear rate of FGM. From Figure 8, it seems that the wear rate of FGM composite materials decreases when increasing the value of *V_fa_* to achieve a minimum value at *V_fa_* = 22%, but then it increases. The wear rate increases when the increase in the volume fraction of fibers is higher than 22%. It is believed that the presence of worn glass fibers on the surface, which act like three-body wear on the contact area to the composites, decreases their ability to resist abrasive wear. Furthermore, from Figure 7 and Figure 8, it can be concluded that the FGM technique can improve the wear rate, but this improvement decreases when increasing the disk speed.

**Figure 9:**
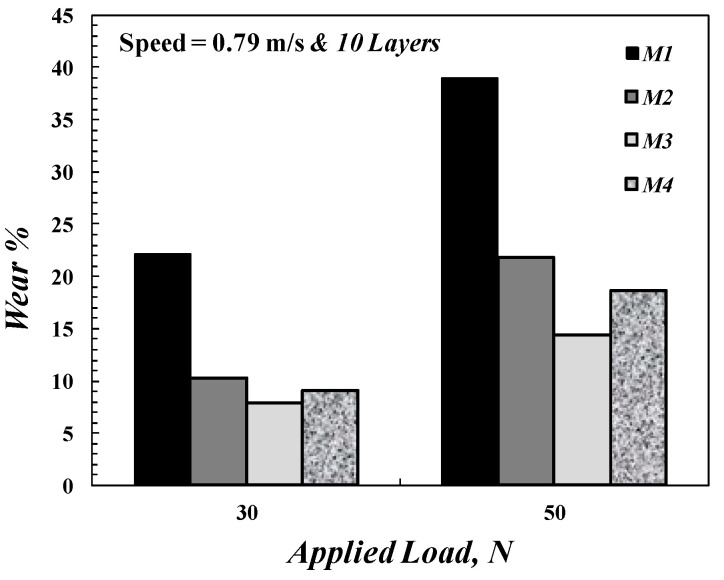
Wear rate as a function of applied load for different FGM specimens.

**Figure 10:**
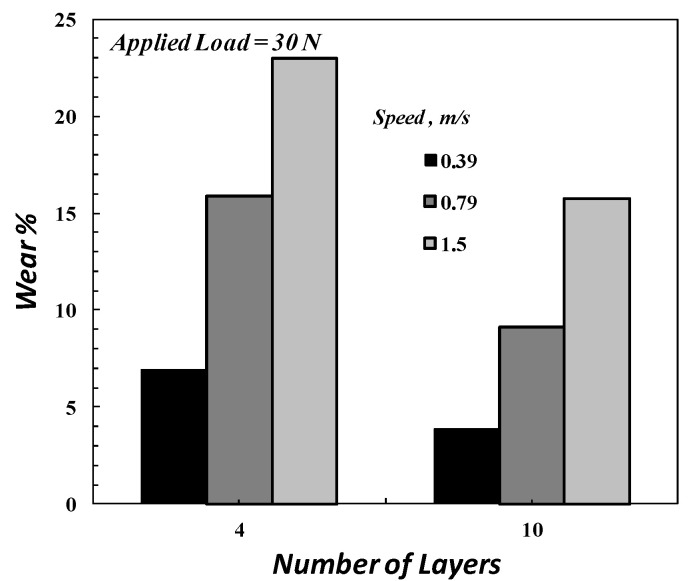
The effect of the number of layers on the wear rate.

The disk speed and applied load are the main factors in the wear test, which affect the wear resistance of composite material, as shown in Figure 7 and Figure 9. Figure 9 shows the wear rate of the FGM specimen for varying applied loads. From Figure 9, it was concluded that the wear rate increases with the applied load; this agrees with [48]. Figure 10 illustrates the effect of the number of composite layers on wear rate by using *M*_4_ and *M*_5_ specimens, which have ten and four layers, respectively. The wear resistance of composite material improves when the number of FGM specimen layers is increased. In other words, the wear rate increases when decreasing the number of the composite layers, as shown in Figure 10; this is because of the increase in the contact area of wear that results from increasing the number of layers of FGM specimens.

**Figure 11:**
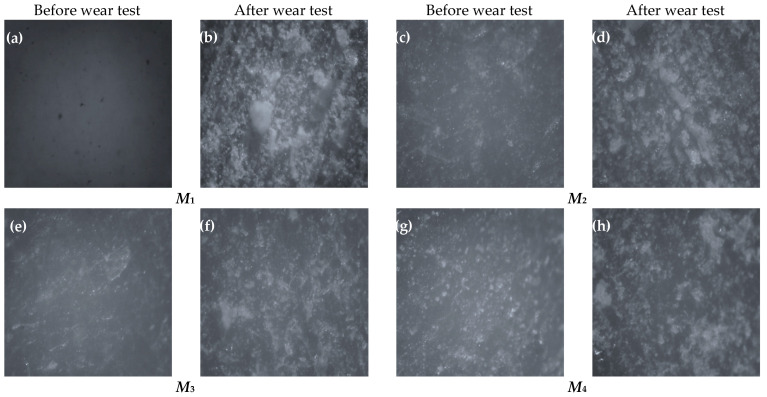

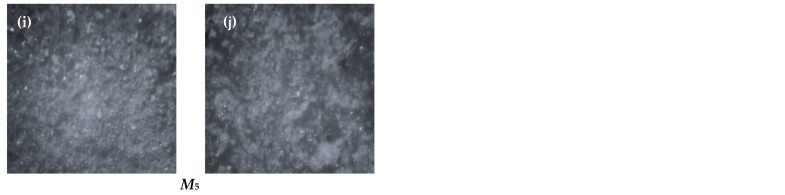
The optical micrographs of the surface of the sample before and after wear tests: (**a**,**b**) *M*_1_, (**c**,**d**) *M*_2_, (**e**,f) *M*_3_, (**g**,**h**) *M*_4_, and (**i**,**j**) *M*_5_.

Figure 11 shows optical micrographs acquired on the surface of prepared specimens before and after the wear test under a normal load of 30 N and at a sliding speed of 0.39 m/s. Figure 11b is the neat epoxy’s optical image, revealing severe wear on the worn surface. After the wear test of the neat epoxy, a deep grove-like scratch formed, and large chunks of the epoxy matrix were damaged and then they peeled off. The worn surfaces of FGM composites specimens (*M*_2_ and *M*_3_) and the conventional composite specimens (*M*_4_ and *M*_5_) are shown in Figure 11c–j. It is noticed that under the same wear test conditions (load and sliding velocity), the FGMs were subjected to mild wear, which is in good agreement with the wear results displayed in Figure 7. As shown in Figure 11c–j, the wear scars are relatively shallow and the peeling of the epoxy on the worn surface is effectively suppressed. The microscopic investigation also reveals that the wear performance of epoxy composites is greatly affected by the number of composite layers and the distribution of fibers inside the epoxy matrix. The ten-layer composite shows a better wear characteristic than the four-layer composite.

**Figure 12:**
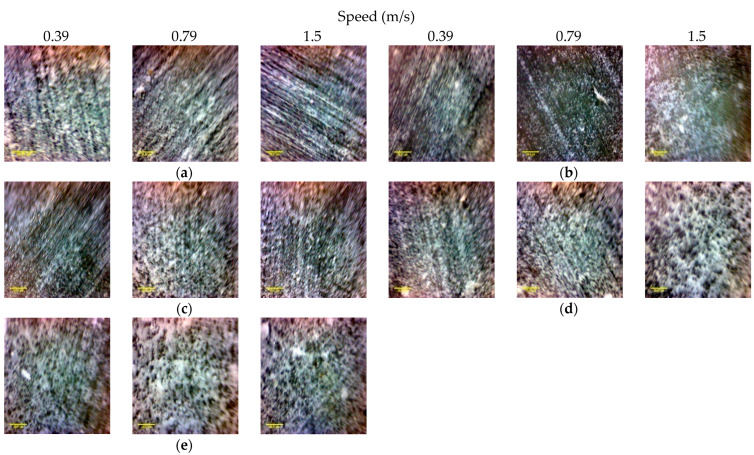
The optical micrographs of the worn surfaces of investigated samples under a normal load of 30 N at various sliding velocities for (**a**) the neat epoxy, *M*_1_, (**b**) *M*_2_, (**c**) *M*_3_, (**d**) *M*_4_, and (**e**) *M*_5_.

**Figure 13:**
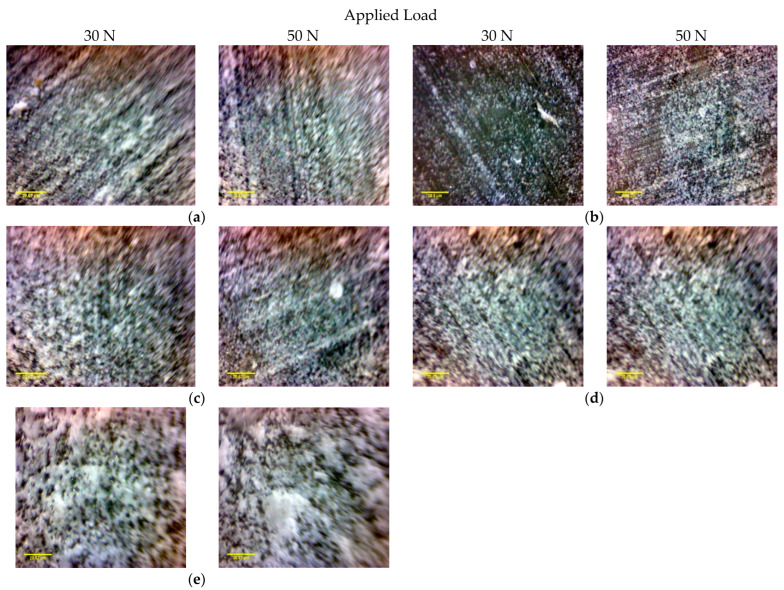
The optical micrographs of the worn surfaces of investigated samples under various normal loads at a sliding velocity of 0.79 m/s for (**a**) the neat epoxy, *M*_1_, (**b**) *M*_2_, (**c**) *M*_3_, (**d**) *M*_4_, and (**e**) *M*_5_.

The wear performance of the neat epoxy and FGM composites are influenced by the normal load and sliding velocity as indicated by microscopic images shown in Figure 12 and Figure 13. Figure 12 and Figure 13 show worn surface variation at various sliding velocities and under different normal loads, respectively. For both neat epoxy and composite specimens, the roughness of the worn surface increases with the increase of the sliding velocity. In addition, with the increase of the sliding velocity, the heat generated, and the surface fatigue at the contact surface may diminish the strength of the specimen, resulting in a rougher surface.

### 4.2. SPT Results

#### 4.2.1. Experimental Results

**Figure 14:**
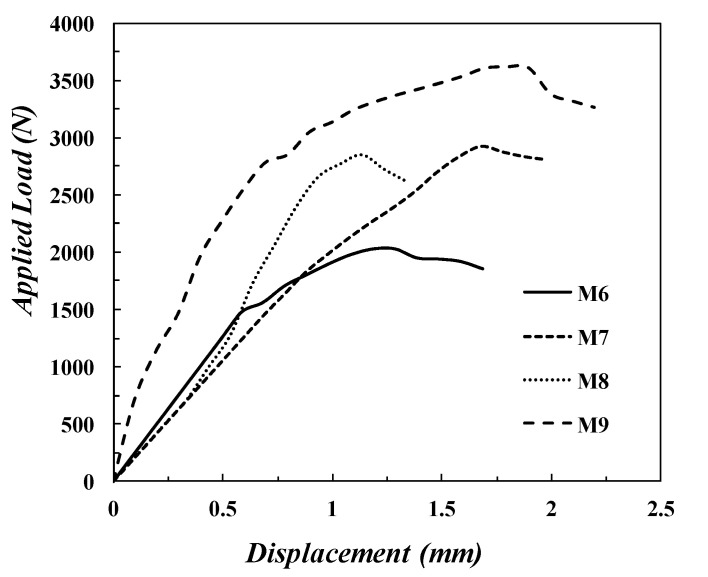
SPT Load–deflection curve for FGM composite samples with four layers varying in each layer glass fiber volume fraction.

Figure 14 shows the load–displacement curve of the FGM specimen, which was described in Table 2 with differing *V_f_* and *V_fa_* under SPT. As is well known from the force-deflection curve recorded during a SP test, the force increases by increasing the deflection and then drops when the specimen fails. Likewise, the present conventional composite specimens (*M*_6_ and *M*_8_) and the FGM specimens (*M*_7_ and *M*_9_) show behavior similar to the public SP test, as shown in Figure 14. Furthermore, from Figure 14, it can be observed that the maximum applied load (ultimate load before failure), *F_m_*, improves by increasing the value *V_fa_* of the conventional specimen, as shown in *M*_6_ and *M*_8_. Regarding specimen *M*_7_, which varies in *V_f_* for each layer and has *V_fa_* of 23.75%, the ultimate load is higher than the conventional specimen *M*_8_, which has *V_fa_* of 30%. This means that varying the arrangement of layers in the composite materials by varying the glass fiber volume of fraction, *V_f_*, for each layer can result in an improvement of the maximum applied load (ultimate load) of the composite material subjected to SPT. In other words, when the distribution of the bundles was according to the FGM, i.e., small bilateral spaces on the surface and larger spaces in the core (a higher *V_f_* in the specimen surface than in the core), the measured parameters in the SPT were improved. Accompanying the specimen, *M*_9_ has higher values of the applied load. From Figure 14, it can be seen that FGM samples have more resistance before the complete fracture of the samples because the presence of the glass fibers with varying distribution helps increase the resistance to failure during the testing.

#### 4.2.2. Numerical Results

##### Modeling Verification

**Figure 15:**
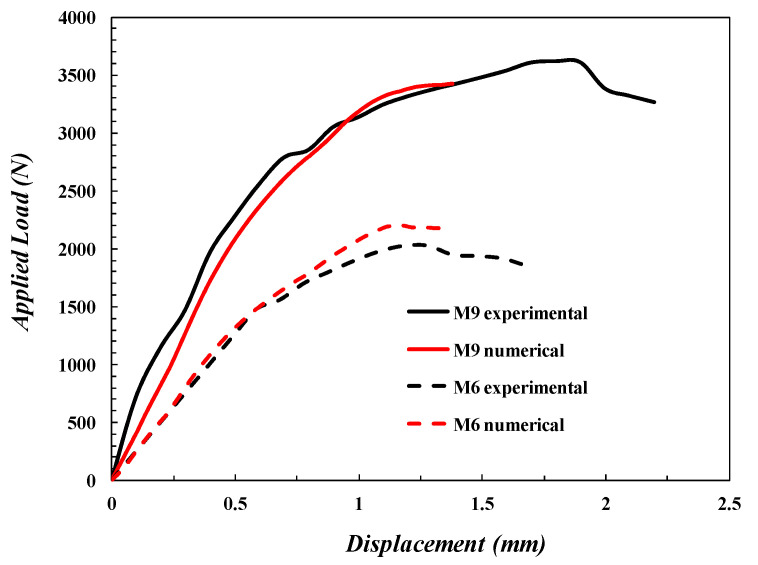
SPT Load–deflection curve for numerical and experimental results for the SPT specimen.

The current numerical results have been compared with experimental results to confirm the current FE model results. Table 6 shows the four specimens’ experimental and numerical maximum loads and their error percentages. The error percentages of the maximum load were less than 10% for all specimens. Figure 15 shows the load–deflection curve of both numerical and experimental values of *M*_6_ and *M*_9_. The figure also shows that both the experimental and numerical tracing had the same behavior. It is clear that Hashin’s failure criterion can predict the damage behavior of composites during the post-peak phase. For more details, see the supplementary materials.

**Figure 16:**
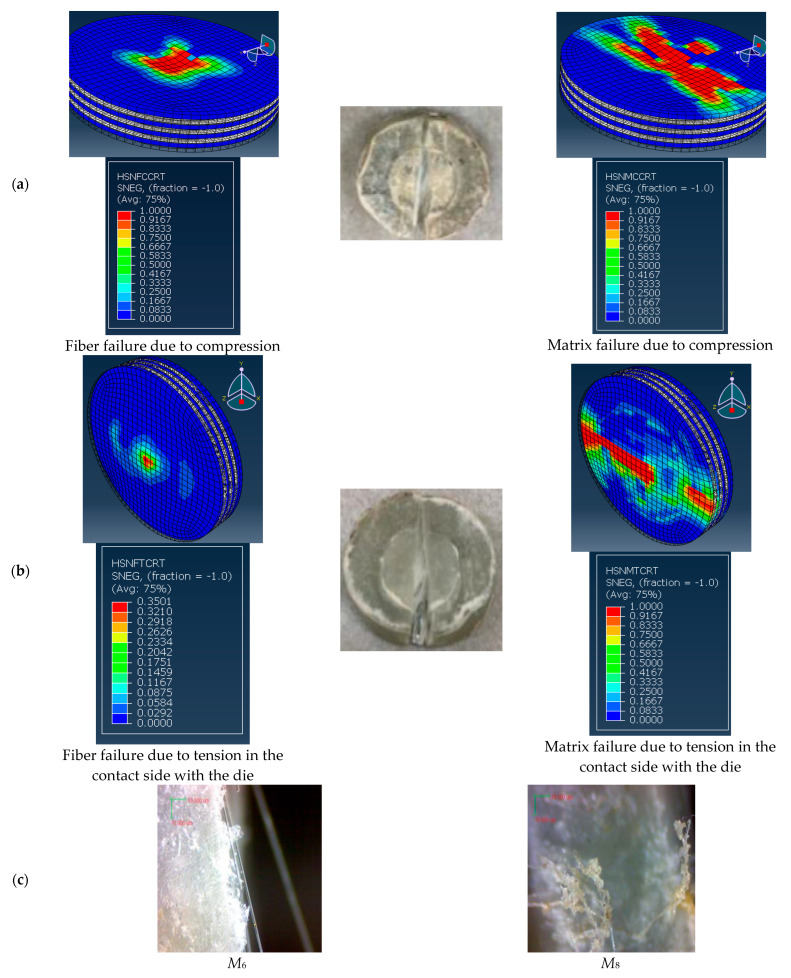
Failure of experimental and numerical of conventional specimens. (**a**) Layer opposite punch (*M*_6_), (**b**) Layer opposite punch (*M*_6_), (**c**) Scan of the specimen.

**Figure 17:**
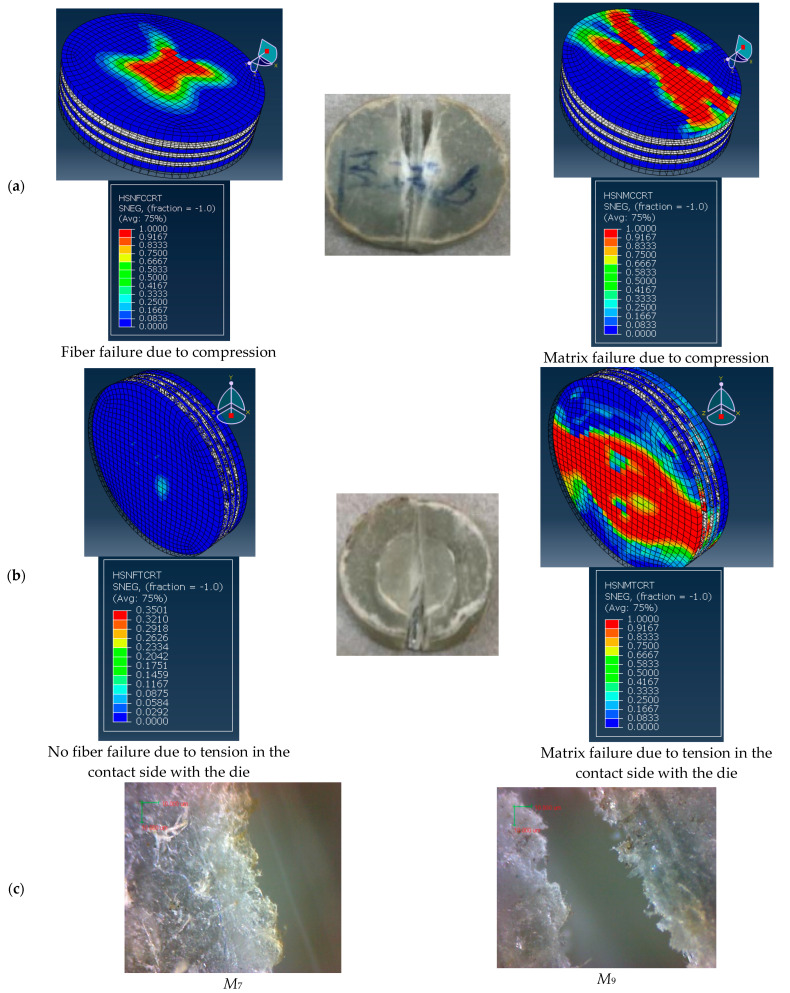
Experimental and numerical failure analysis of FGM specimens. (**a**) Layer opposite punch (*M*_7_), (**b**) Layer opposite punch (*M*_7_), (**c**) Scan of the specimen.

The numerical and experimental final failure of conventional and FGM specimens subjected to SPT can be observed in Figure 16 and Figure 17, respectively. In the present work, Hashin’s failure criteria associated with CZM were implemented to predict the fiber and matrix damage due to tensile or compression stress and delamination between the layers, as shown in Figure 16 and Figure 17 for conventional and FGM specimens. The experimental and numerical results for all specimens showed that the fracture of the specimen was parallel to the fiber direction, as shown in Figure 16c and Figure 17c. Figure 16 shows the failure of the layer opposite the punch tip, while Figure 16b shows the failure in the layer in contact with the die for the conventional composite specimen, *M*_6_. Figure 16c shows the scanning of the fracture edge for specimens *M*_6_ and *M*_8_, which showed the unbroken long fibers. The other traditional specimen, *M*_8_, had the same behavior. Figure 17a shows the failure of the layer opposite the punch tip, while Figure 17b shows the failure in the layer in contact with the die for the FGM composite specimen *M*_9_. Figure 17c shows the scanning of the fracture edge for specimens *M*_7_ and *M*_9_, which showed the unbroken long fibers. The other FGM specimen, *M*_7_, had the same result.

From Figure 16 and Figure 17, it can be concluded that the final damage of the conventional and FGM specimens began along and beneath the punch tip and grew along the direction of the fiber; after that, the specimen broke into two pieces. Therefore, the fiber orientation is the main factor that can affect the shape of the final damage of the composite material under SPT. The cross-ply or the woven composite material may improve the resistance to fracturing.

Therefore, the verification of the present FE model, which was illustrated in Table 5 and Figure 15, Figure 16 and Figure 17, proves that the present numerical results can be accepted.

##### The Parametric Study

**Figure 18:**
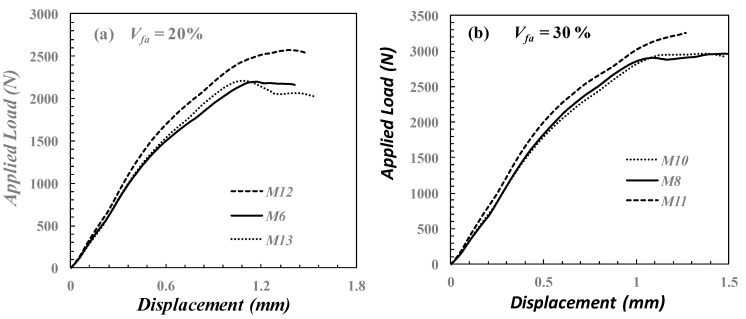
Numerical load–deflection curve of FGMs specimen with: (**a**) *V_fa_* of 20% and (**b**) *V_fa_* of 30%.

As described in the experimental section, the FGM technique can improve the flexural strength of conventional composite specimens measured from SPT. However, it needs further investigation. Therefore, four additional specimens with varying *V_f_* for each layer were used, as described in Table 3. Figure 18a,b illustrate the comparison of the conventional composite configuration and the FGM specimen for *V_fa_* = 20% and 30% subjected to SPT, respectively. Figure 18a shows that the specimen with a higher *V_f_* for the outer layer, *M*_12_, has a maximum applied load greater than that of the conventional specimen, *M*_6_, and higher than the specimen with a higher *V_f_* of the inner layer, *M*_13_. In addition, the specimen that has a higher *V_f_* in the outer layer has the maximum applied load, as shown in Figure 18b. From Figure 14 and Figure 18, it can be concluded that the *V_f_*% of the outer layers and the average volume fraction of the specimens are major factors in improving their strength, as is confirmed with the results obtained in refs. [31,49].

**Figure 19:**
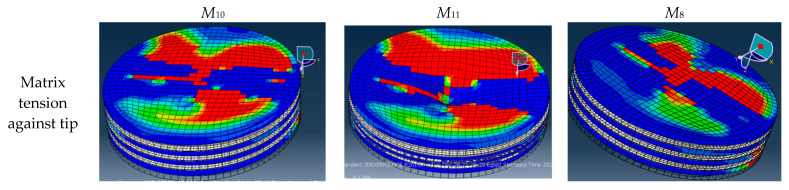

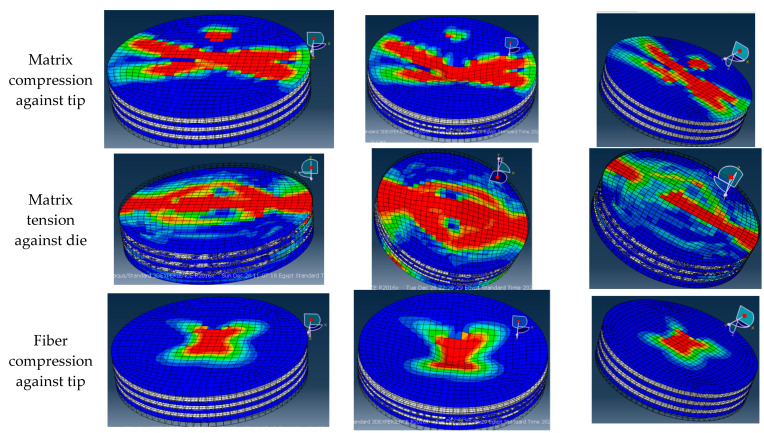
Comparison between the 30% V_f_% ordinary and FGM (specimens *M*_8_, *M*_10_, and *M*_11_) failure elements.

Figure 19 shows a comparison between the failure profiles of the three specimens *M*_8_, *M*_10_, and *M*_11_, with their load deflection curves shown in Figure 18b. The figure shows that all the three specimens have the same failure shape, while the specimen that has the largest load value, *M*_11_, has a higher percentage of damaged elements on the outer layers. From the figure it can be concluded that the main reason for the specimen failure was the matrix compression failure in the upper layer, against the tip layer, and the matrix tension failure in the lower layer, against the die layer. These two failures coincided with the x-axis, which is the direction in which the fibers are oriented.

## 5. Conclusions

This study evaluates the mechanical and tribological behavior of conventional and FG polymeric composites reinforced with continuous glass fibers. The main findings are summarized as follows:

- The wear resistance of the composites increases by increasing the average fiber volume fraction up to 22%. An increase in the number of layers helps improve the wear resistance of composite materials.
- The existence of worn glass fibers on the surface acts like three-body wear on the contact area and decreases the composite’s ability to resist abrasive wear.
- SPT results showed that the ultimate load of FG composites increased by 30% compared with the conventional composites.
- The *V_f_* % of the outer layers and the average volume fraction of SPT specimens are the major factors in improving their flexural strength.
- The final damage of the polymeric composites reinforced with continuous glass fibers specimens under SPT starts beneath the punch tip and grows in the direction of the fiber, after which the specimen is broken into two pieces.
- The results were confirmed by modeling the SPT test. This allows additional studies for more specimen configurations to study the advantages of the FGM over conventional composite materials.
- A comparison of standard sandwich structures and FGM patterns, in particular, the pull-out strength of the inserts embedded in them, will be of interest for study in future work.

## Figures and Tables

**Table 1:** Mechanical properties of E-glass fiber and epoxy resin.

Mechanical Properties	E-Glass Fiber	Epoxy
Modulus of elasticity, *E*, (GPa)	72.4	2–6
Tensile strength (MPa)	3450	35–130
Elongation %	1.8–3.2	1–8.5
Poisson’s ratio, ν,	0.22	0.36

**Table 2:** The layers’ *V_f_* values for the different specimens with average *V_fa_*.

Test	Average *V_fa_*	Symbol	Layer *V_f_* (%)									
			1	2	3	4	5	6	7	8	9	10
Wear	0%	*M*_1_	0	0	0	0	0	0	0	0	0	0
	31%	*M*_2_	50	50	20	20	15	15	20	20	50	50
	22%	*M*_3_	30	30	17.5	17.5	15	15	17.5	17.5	30	30
	22%	*M*_4_	22	22	22	22	22	22	22	22	22	22
	22%	*M*_5_	22	22	22	22	-	-	-	-	-	-
SPT	20%	*M*_6_	20	20	20	20	-	-	-	-	-	-
	23.75%	*M*_7_	30	17.5	17.5	30	-	-	-	-	-	-
	30%	*M*_8_	30	30	30	30	-	-	-	-	-	-
	35%	*M*_9_	50	20	20	50	-	-	-	-	-	-

**Table 3:** The layers’ *V_f_* values for the different specimens with average *V_fa_*.

Test	Average *V_fa_*	Symbol	Layer *V_f_* (%)			
			1	2	3	4
SPT	30%	*M*_10_	40	20	20	40
	30%	*M*_11_	50	10	10	50
	20%	*M*_12_	30	10	10	30
	20%	*M*_13_	10	30	30	10

**Table 4:** Lamina’s elastic constants for different *V_f_*% values.

*V_f_* (%)	Density (gm/cm^3^)	Young’s Modulus (GPa)			In-Plane Shear Modulus (GPa)			Poisson’s Ratio		
		*E_X_*	*E_Y_*	*E_Z_*	*G_XY_*	*G_XZ_*	*G_YZ_*	*υ_xy_*	*υ_xz_*	*υ_yz_*
50	1.825	38.2936	8.8788	8.8788	3.4521	3.4521	3.0432	0.277	0.277	0.458
40	1.68	31.3283	6.8910	6.8910	2.7656	2.7656	2.3042	0.290	0.290	0.495
30	1.535	24.3614	5.6323	5.6323	2.2530	2.2530	1.8643	0.304	0.304	0.510
20	1.39	17.3928	4.7922	4.7922	1.8557	1.8557	1.5908	0.318	0.318	0.506
17.5	1.354	15.6504	4.6259	4.6259	1.7700	1.7700	1.5403	0.322	0.322	0.501
10	1.245	10.4224	4.1925	4.1925	1.5388	1.5388	1.4220	0.3339	0.3339	0.4741

**Table 5:** Hashin’s damage model strength data for various *V_f_*% values.

*V_f_*%	Longitudinal Tensile Strength *X^T^* (MPa)	Transverse Tensile Strength *Y^T^* (MPa)	Longitudinal Compressive Strength *X^C^* (MPa)	Transverse Compressive Strength *Y^C^* (MPa)	Longitudinal Shear Strength *S^L^* (MPa)	Transverse Shear Strength *S^T^* (MPa)
50	1806.4124	44.8321	489.8681	111.3933	65.000	55.696
40	1477.6949	50.6262	488.9371	97.7584	65.0000	48.8792
30	1148.9774	56.3602	457.5301	87.3665	65.000	43.6833
20	820.2599	62.7957	393.7940	79.0956	65.000	39.5478
17.5	738.0805	64.6491	372.3337	77.8411	65.000	38.9205
10	491.5424	71.4214	292.8034	72.5700	65.0000	36.2850

**Table 6:** Experimental and numerical maximum applied load (N).

Specimen Code	Max. Load, *F_m_*		
	Experimental	Numerical	Error %
*M*_6_	2026.263	2201.95	8.670508
*M*_7_	2929.286	2662.27	−9.1154
*M*_8_	2852.661	2862.4	0.341386
*M*_9_	3624.024	3420.41	−5.61845

## Data Availability

All the data required are reported in this manuscript and supplementary materials.

## Supplementary Materials

The following supporting information can be downloaded at: https://www.mdpi.com/article/10.3390/polym14102057/s1, Sec-1: Mechanical properties of glass fiber-epoxy lamina; Sec-2: Hashin Model Constants; Sec-3: Hashin Damage Criteria; Computer Code: Computer Code.inp.
