# Supplementary material for: Mechanical and Tribological Behavior of Functionally Graded Unidirectional Glass Fiber-Reinforced Epoxy Composites
Source: Polymers (Basel). 2022 May 18;14(10):2057. doi: 10.3390/polym14102057 (PMC9147567; doi:10.3390/polym14102057)
Supplement: Supplementary file 1 [file polymers-14-02057-s001.zip › polymers-1710300-supplementary.pdf]

Supplementary Materials: Mechanical and Tribological Behavior of Functionally Graded Unidirectional Glass Fiber-Reinforced Epoxy Composites

W.H. Alhazmi[a], Y. Jazaa[b], S.M. Althahban[a], S. Mousa[a], A. Abu-Sinna[c], A.A. Abd-Elhady[d], H.E.M. Sallam[e], M. Atta[e]

[a]Faculty of Engineering, Jazan University, Jazan 706, Kingdom of Saudi Arabia. E-mail: waalhazmi@jazanu.edu.sa, salthahban@gmail.com, samousa@jazanu.edu.sa
[b]Faculty of Engineering, King Khalid University, Kingdom of Saudi Arabia. E-mail: yjazaa@kku.edu.sa
[c]Force and Material Metrology Department, National Institute of Standards, Giza, Egypt. E-mail: a_abu_sinna@yahoo.com

[d]Mechanical Design Dept., Faculty of Engineering, Helwan University, Cairo 11718, Egypt. E-mail: amrabdelfattah@m-eng.helwan.edu.eg
[e]Faculty of Engineering, Zagazig University, Zagazig, Egypt. E-mail: hem_sallam@zu.edu.eg, matta767@gmail.com

Sec-1. Mechanical properties of glass fiber-epoxy lamina

Lamina's elastic constants for different of unidirectional fiber GFRP calculations can be calculated analytically by using the Equations S1 to S11 according to its V_f ratios [1,2].

Density (V_f * $_f$ + V_m * $_m$ gm/cm^3 (S1)

$$E_{11} = V_f E_f + V_m E_m + \frac{4 * V_f V_m (\vartheta_f - \vartheta_m)^2}{\frac{V_f}{K^m} + \frac{1}{G^m} + \frac{V_m}{K^f}} \quad \text{(S2)}$$

$$K_f = \frac{E_f}{2(1-2\vartheta_f)(1+\vartheta_f)}. \quad \text{(S3)}$$

$$K_m = \frac{E_m}{2(1-2\vartheta_m)(1+\vartheta_m)}. \quad \text{(S4)}$$

$$E_{22} = E_{33} = 2(1 + \vartheta_{23}) G_{23} \quad \text{(S5)}$$

$$G_{12} = G_{13} = G_m \frac{G_f(1+V_f) + G_m V_{m)}}{G_f V_f + G_m(1+V_f)} \quad \text{(S6)}$$

G_{23} is calculated with the solving of the Eq.S7 (*A*, *B* and *C* are Equations defined in ref. [4]).

$$A(\frac{G_{23}}{G_m})^2 + 2B\left(\frac{G_{23}}{G_m}\right) + C = 0 \quad \text{(S7)}$$

$$\vartheta_{12} = \vartheta_{13} = V_f\vartheta_f + V_m\vartheta_m + \frac{V_f V_m(\vartheta_f - \vartheta_m)(\frac{1}{K_m} - \frac{1}{K_f})}{\frac{V_f}{K^m} + \frac{1}{G^m} + \frac{V_m}{K^f}} \quad \text{(S8)}$$

$$\vartheta_{23} = \frac{K - mG_{23}}{K + mG_{23}} \quad \text{(S9)}$$

$$m = 1 + 4K\frac{\vartheta_{12}^2}{E_{11}} \quad \text{(S10)}$$

$$K = \frac{K_m(K_f + G_m)V_m + K_f(K_m + G_m)V_f}{(K_f + G_m)V_m + (K_m + G_m)V_f} \quad \text{(S11)}$$

Sec-2. Hashin Model Constants

Hashin's damage model strength data can be calculated analytically by using the Equations S12 to S16 according to its V_f.ratios [1,2]. S^L is set to 65 MPa for all the specimens, as the average value for all V_f ratios of unidirectional Fiber Reinforcement Plastic based on experiments [3].Note that the transverse shear strength (S^T) is half of Y^C values [4].

$$X^T = \sigma_f V_f + \sigma_m' (1 - V_f) \quad \text{(S12)}$$

$$Y^T = \frac{\sigma_m}{K_\sigma} \quad \text{(S13)}$$

$$X^C = \frac{\lfloor E_f V_f + E_m(1 - V_f)\rfloor \left(1 - V_f^{\frac{1}{3}}\right) \epsilon_{mu}^c}{\vartheta_f V_f + \vartheta_m(1 - V_f)} \quad \text{(S14)}$$

$$Y^C = E_{22}[\frac{d}{s} * \frac{E_m}{E_{22}} + \left(1 - \frac{d}{s}\right)]\epsilon_{mu}^c \quad \text{(S15)}$$

Sec-3. Hashin Damage Criteria

The initiation damage mechanisms considered in Hashin's damage model are tension and compression failure of the fiber and matrix. Equations S16 to S21 describe the general forms controlling these mechanisms.

Fiber damage mechanisms;

If $(\hat{\sigma}_{11} \geq 0\,,\ tension)$ $\quad F_f^t = (\frac{\hat{\sigma}_{11}}{X^T})^2 + \alpha(\frac{\hat{\tau}_{12}}{S^L})^2$ (S16)

If $(\hat{\sigma}_{11} < 0\,,\ comperssion)$ $\quad F_f^c = (\frac{\hat{\sigma}_{11}}{X^C})^2$ (S17)

Matrix damage mechanisms;

If $(\hat{\sigma}_{22} \geq 0\,,\ tension)$ $\quad F_m^t = (\frac{\hat{\sigma}_{22}}{Y^T})^2 + \alpha(\frac{\hat{\tau}_{12}}{S^L})^2$ (S18)

If $(\hat{\sigma}_{22} < 0\ ,\ compersssion)$ $\quad F_m^c = (\frac{\hat{\sigma}_{22}}{2S^T})^2 + [(\frac{Y^C}{2S^T})^2 + 1]\frac{\hat{\sigma}_{22}}{Y^C} + (\frac{\hat{\tau}_{12}}{S^L})^2$ (S19)

$$\begin{bmatrix} \hat{\sigma}_{11} \\ \hat{\sigma}_{22} \\ \hat{\tau}_{12} \end{bmatrix} = \begin{bmatrix} \frac{1}{1-d_f} & 0 & 0 \\ 0 & \frac{1}{1-d_m} & 0 \\ 0 & 0 & \frac{1}{1-d_s} \end{bmatrix} \begin{bmatrix} \sigma_{11} \\ \sigma_{22} \\ \tau_{12} \end{bmatrix} \quad \text{(S20)}$$

$$d_s = 1 - (1 - d_f^t)(1 - d_f^c)(1 - d_m^t)(1 - d_m^c) \quad \text{(S21)}$$

The initial values of d_f, d_m,, and d_s are set to zero (since the effective stresses are equal to the true stresses of the material) and are increased with the loading progress until reaching one. Through the increase of the load, the effective strength of the elements is reduced according to Eq. S20. The elements that have a damage variable equal toone areexcluded, as these elements do not endure this type of stress any longer. Throughout the present work, α is set to zero, which means that the contributionof the shear stress in the fiber initiation damage is neglected.

**Where;**	
$\hat{\sigma}_{11}, \hat{\sigma}_{22}$ and $\hat{\tau}_{12}$	Effective stresses in X direction (fiber direction) , Y direction and X–Y plan respectively.
X^T	Longitudinal tensile strength (MPa).
X^C	Longitudinal compressive strength (MPa).
Y^T	Transverse tensile strength (MPa).
Y^C	Transverse compressive strength (MPa).
S^L	Longitudinal shear strength (MPa).
S^T	Transverse shear strength (MPa).
α	Coefficient of shear stress contribution in the fiber initiation damag
d_f^t	Tension fiber damage variable.
d_f^c	Compression fiber damage variable.
d_m^t	Tension matrix damage variable.
d_m^c	Compression matrix damage variable.
d_s	Shear damage variable.
ρ	Density (gm/cm^3).
V_f	Fiber volume fraction (%).
V	Volume fraction.
d	Fiber diameter.
E_{11}, E_{22} and E_{33}	Young’s modulus in X direction (fiber direction) , Y direction and Z direction respectively.

G_{12} and G_{13}	Shear modulus in X–Y plan and X–Z plan respectively.
ϑ_{12} , ϑ_{23} and ϑ_{13}	Poisson's ratio in X–Y plan, Y–Z plan and X–Z plan respectively.
f	Mean fiber.
m	Mean matrix.
s	Distance between fiber
ϵ_{mu}	Fracture tension strain of matrix. .
ϵ_{mu}^{c}	Fracture compression strain for matrix.

References

[1] M Atta, A Abu-Sinna, S Mousa, HEM Sallam and AA Abd-Elhady, "Flexural behavior of functionally graded polymeric composite beams", Journal of Industrial Textiles (2021), 0(0). https://doi.org/10.1177/15280837211000365

[2] Islam El-Sagheer, Amr A Abd-Elhady, Hossam El-Din M Sallam and Soheir A R Naga, "An Assessment of ASTM E1922 for Measuring the Translaminar Fracture Toughness of Laminated Polymer Matrix Composite Materials", Polymers 2021, 13, 3129, DOI: https://doi.org/10.3390/polym13183129

[3] Rafiee R., Amini A., "Modeling and experimental evaluation of functional failure pressures in glass fiber reinforced polyester pipes", Computational Materials Science, 96, 2015, 579–588

[4] ABAQUS 6.14, Analysis User's Manual, vol. 5, (2014) (© Dassault Systems).
